# Covalently Linking Oligomerization-Impaired GlpF Protomers Does Not Completely Re-establish Wild-Type Channel Activity

**DOI:** 10.3390/ijms20040927

**Published:** 2019-02-20

**Authors:** Noreen Klein, Margareta Trefz, Dirk Schneider

**Affiliations:** Johannes Gutenberg-University Mainz, Institute of Pharmacy and Biochemistry, Johann-Joachim-Becher-Weg 30, 55128 Mainz, Germany; noreen-klein@freenet.de (N.K.); martrefz@uni-mainz.de (M.T.)

**Keywords:** aquaglyceroporin, covalent linkage, GlpF, homooligomer, membrane protein, protein folding, tetramer, interaction

## Abstract

Integral membrane proteins of the aquaporin family facilitate rapid water flux across cellular membranes in all domains of life. Although the water-conducting pore is clearly defined in an aquaporin monomer, all aquaporins assemble into stable tetramers. In order to investigate the role of protomer–protomer interactions, we analyzed the activity of heterotetramers containing increasing fractions of mutated monomers, which have an impaired oligomerization propensity and activity. In order to enforce interaction between the protomers, we designed and analyzed a genetically fused homotetramer of GlpF, the aquaglyceroporin of the bacterium *Escherichia coli* (*E. coli*). However, increasing fractions of the oligomerization-impaired mutant GlpF E43A affected the activity of the GlpF heterotetramer in a nearly linear manner, indicating that the reduced protein activity, caused by the introduced mutations, cannot be fully compensated by simply covalently linking the monomers. Taken together, the results underline the importance of exactly positioned monomer–monomer contacts in an assembled GlpF tetramer.

## 1. Introduction

**Figure 1:**
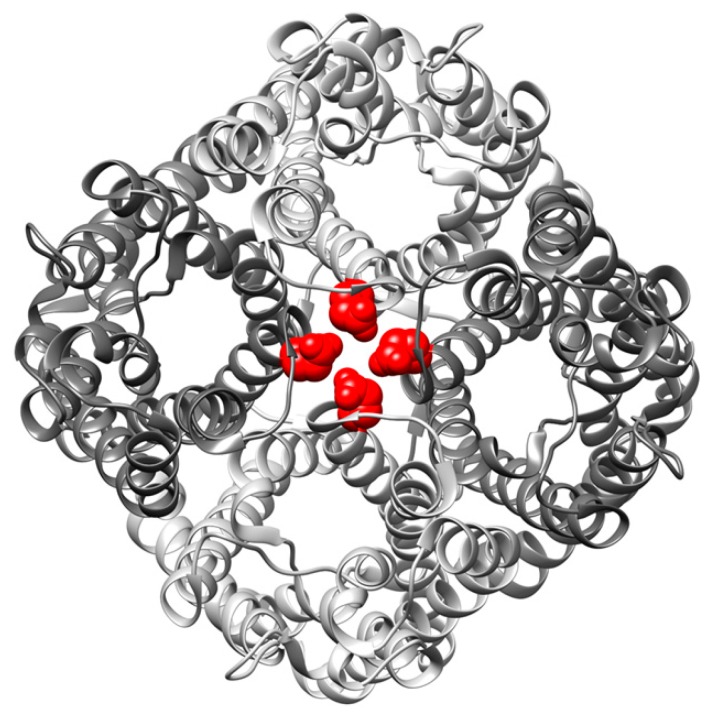
Top view on a GlpF tetramer with Glu 43 highlighted. The GlpF tetramer consists of four GlpF monomers (shown in different gray tones) each with a glycerol-conducting pore. The residue Glu 43 (red) is located in the central pore of the GlpF tetramer (PDB-ID: 1FX8).

Aquaporins are highly selective polytopic transmembrane (TM) channel proteins that facilitate the flux of water across cellular membranes. While in the bacterium *E. coli* only the classical aquaporin AqpZ and the glycerol facilitator GlpF are expressed, in humans 13 aquaporins (AQPs) have been identified thus far and for some plants up to 30 AQPs are described [1,2]. In addition to facilitating water flux, the subfamily of the aquaglyceroporins facilitates the flux of small polar substrates, such as the linear polyalcohol glycerol. Permeability for substrates, like urea, nitrate, ammonia, hydrogen peroxide, arsenite, silicate, antimonite and even ions has also been described [3,4,5]. The AQP translocation pore is defined in the monomer (Figure 1), and thus, AQPs are facultative oligomers [6]. AQPs assemble into stable homotetramers in vivo and in vitro, resulting in formation of an additional fifth pore in the center of the tetramer [7,8,9,10,11,12]. The formation of this additional pore, which possibly allows the flux of gaseous substrates such as CO_2_ and NO across the lipid bilayer, is suggested to be a driving force for AQP tetramerization [13,14]. However, the flux of gaseous substrates through the central pore has not been sufficiently demonstrated and is still controversially discussed [15]. Nonetheless, AQP tetramerization appears to be essential for the stability and function of AQPs, as the GlpF mutant E43A has an impaired oligomerization propensity coupled with an impaired activity [16]. The respective Glu residue is not a part of the substrate-conducting pore, but is positioned in the GlpF transmembrane (TM) helix 2 which resides at the monomer–monomer interface where it potentially drives oligomerization via strong hydrogen bond formation [17,18,19,20,21].

AQP tetramerization might also contribute to an increased in vivo stability, as shown for the *E. coli* aquaglyceroporin GlpF [16]. Moreover, a positive cooperativity is indicated for water conductance in an assembled AQP tetramer [22,23,24], and genetic fusion of two water-conducting NtPIP2;1 and two non-water-conducting NtAQP1 monomers from *Nicotiana tabacum* resulted in a water conductance rate resembling a homotetramer consisting of solely water-conducting NtPIP2;1 monomers [25]. These observations suggest that conformational changes, induced by interaction of the protomers within the heterotetramer, enable water conductance of the otherwise non-water-conducting NtAQP1 monomers [25].

Thus, several recent observations indicate that AQP tetramers are not simply an assembly of functional AQP monomers; rather, tetramerization appears to be crucial for the channel’s conductance. To gain more information about the role of AQP tetramerization, we designed GlpF tetramers consisting of increasing proportions of interaction-impaired E43A-mutated monomers and constructed a genetically fused homotetramer of the *E. coli* aquaglyceroporin GlpF. Via this approach we tested whether forcing individual monomers into close proximity can compensate for the impaired tetramerization and re-establish protein activity in vivo.

Enforced interaction of wild-type (WT) and E43A-mutated monomers within a fused GlpF tetramer could not completely re-establish WT activity in the produced heterotetramer. This suggests that the monomer activity critically depends on correct non-covalent interactions with adjacent protomers and that the decreased activity of the E43A mutant cannot simply be neutralized by enforcing monomer interactions via covalent linkage.

## 2. Results and Discussion

**Figure 2:**
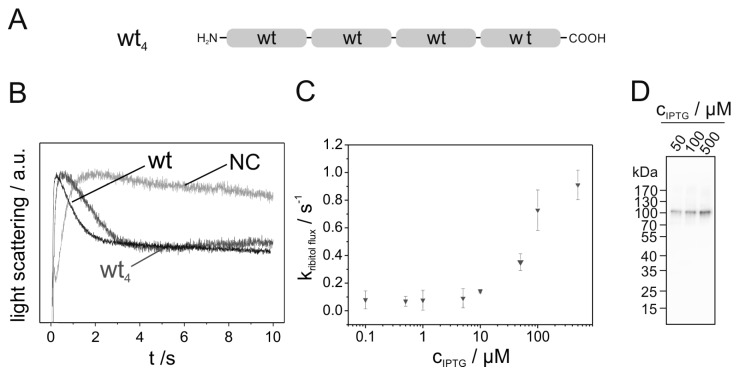
Activity of genetically fused GlpF homooligomer. (**A**) Schematic representation of the fused GlpF wild-type (WT) homooligomer analyzed. Each light gray box represents one WT GlpF. (**B**) Typical light- scattering curves of the WT_4_ GlpF homooligomer (dark gray) and the (non-fused) GlpF WT (black), determined after induction of protein expression with 500 µM IPTG. GlpF-free control cells (NC) (gray) were transformed with the plasmid pMalp2 and treated exactly as the protein expressing cells. The light-scattering curves are the average of five measurements. (**C**) The rate constants of the ribitol conductance, facilitated by the fused GlpF WT homotetramer (WT_4_) (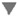), were determined by approximation of the light-scattering curves using a single exponential decay function (*n* = 3 ± SD) (see also Figure S1). (**D**) Western blot analysis, determining the expression level of the studied GlpF homooligomer at increasing isopropyl β-D-1-thiogalactopyranoside (IPTG) concentrations. For the Western blot analysis, an antibody recognizing the GlpF C-terminus was used.

Recent results have indicated that interactions of individual GlpF monomers within a tetramer are crucial for the activity of the protein, albeit the channel pore is formed by a single GlpF monomer. As GlpF is a facultative oligomer (i.e., the monomeric protein contains the active channel) [26], this observation has raised the question whether the GlpF activity might be increased in the tetramer due to an inter-protomer stabilization of the individual channels located within each of the four GlpF monomers. In the present study, we enforced close proximity of GlpF monomers by expressing a genetically fused GlpF WT tetramer (WT_4_, Figure 2A). Since the protein’s C- and N-termini are both located at the cytoplasmic side of the membrane, the orientation of the monomers remained preserved upon fusion.

To test whether the engineered GlpF homotetramer was properly expressed, *E. coli* membranes were isolated and the amount of WT_4_ protein present in the membranes was assessed immunochemically via Western blot analysis. Genetic fusion of four GlpF monomers resulted in expression of a synthetically fused GlpF tetramer, having a calculated molecular mass of 120 kDa (Figure 2D).

To analyze the activity of WT_4_, kinetics of GlpF-mediated ribitol flux across the inner *E. coli* membrane were determined [9]. Rapid mixing of GlpF expressing *E. coli* SK46 cells with a hypertonic ribitol solution causes initial cell shrinkage, owing to water efflux. As ribitol diffuses into the cells via GlpF, water flows back causing the cells to re-swell. Shrinkage and re-swelling of the cells can be assessed via measuring the light-scattering intensity, as it causes an initial increase followed by a decay of the light-scattering signal (Figure 2B). As can be seen in Figure 2B, the ribitol conductance of the fused GlpF tetramer was slightly decreased compared to the non-fused GlpF WT, yet the WT_4_ protein was still highly active. The slightly reduced activity of the WT_4_ homooligomer in comparison to the non-fused WT GlpF might have been caused by a reduced flexibility of the GlpF N- and/or C-terminus corresponding to recent observations in yeast AQPs [27]. To assess the rate constants of GlpF-facilitated ribitol flux (Figure 2C), the decay was fitted with a single exponential function (see also Figure S1). When the amount of expressed protein was increased by increasing the amount of isopropyl β-D-1-thiogalactopyranoside (IPTG) added to *E. coli* cells (Figure 2D and Figure S2), significant protein expression was observed starting at 50 µM IPTG coupled with an increased ribitol conductance rate (Figure 2C).

**Figure 3:**
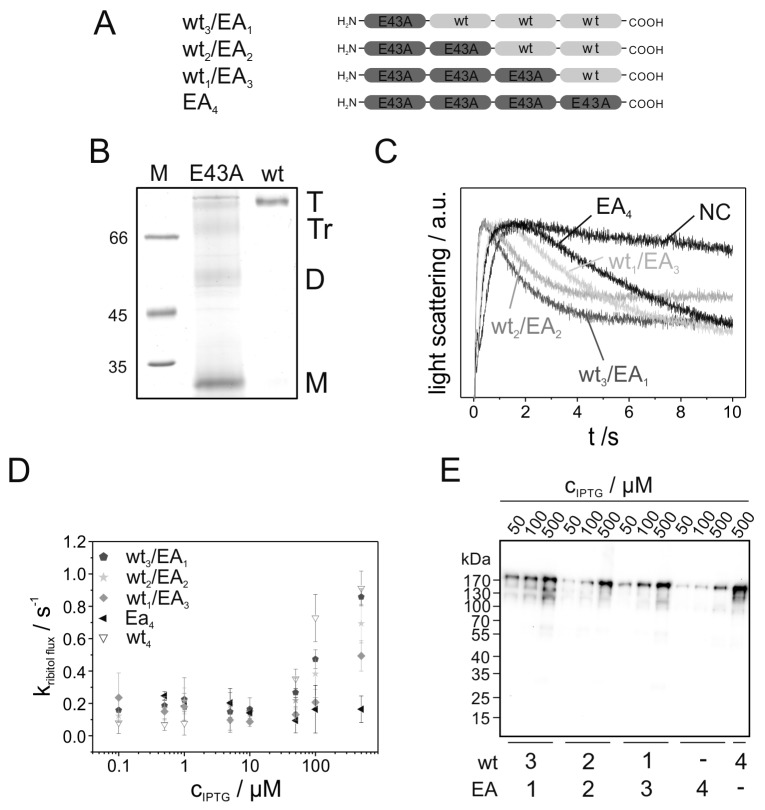
Activity of genetically fused GlpF heterotetramers. (**A**) Schematic representation of the fused GlpF oligomers. The light gray and dark gray boxes represent WT and E43A-mutated GlpF, respectively. (**B**) SDS-PAGE gel showing detergent-solubilized and purified GlpF WT as well as the oligomerization-impaired GlpF E43A. Purification and SDS-PAGE analysis were performed as described in [16]. A 10% SDS gel was used and no additional SDS has been added to the sample buffer. The GlpF tetramer (T) shows a band at ~100 kDa whereas the band of the monomeric GlpF (M) is visible at ~30 kDa. Additionally, bands representing a GlpF dimer (D) and trimer (Tr) are visible for the mutant. (**C**) Typical light-scattering curves of the fused GlpF tetramers WT_3_/EA_1_ (dark gray), WT_2_/EA_2_ (light gray), WT_3_/EA_1_ (middle gray) and EA_4_ (black) after induction of protein expression with 500 µM IPTG. GlpF-free control cells (NC) (gray) were transformed with the plasmid pMalp2 and treated exactly as GlpF-expressing cells. The light-scattering curves are the average of five measurements. (**D**) The rate constants of ribitol conductance facilitated by the GlpF tetramers WT_3_/EA_1_ (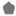), WT_2_/EA_2_ (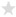), WT_1_/EA_3_ (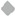) and EA_4_ (◀) were determined by approximation of the light- scattering curves using a single exponential decay function (*n* = 3 ± SD). For comparison, the rate constants of the ribitol flux facilitated by the fused tetramer WT_4_ (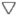) are also depicted. (**E**) Western blot analysis, determining the expression level of the various fused GlpF tetramers. For the Western blot analysis, an antibody recognizing the GlpF C-terminus was used.

To glean information about a possible interaction between individual GlpF monomers within a tetrameric assembly, genetically fused GlpF tetramers with increasing amounts of the tetramerization-impaired E43A-mutated monomers were constructed (Figure 3A). Introducing the E43A mutation in the (non-fused) GlpF WT monomer reduced the protein activity by approximately 30% [16]. Purified (non-linked) GlpF E43A had a reduced tetramer stability and was mainly visible as a monomer band on SDS gels (Figure 3B). The activity of the fused heterotetramers carrying increasing numbers of mutated monomers was assessed by measuring the ribitol conductance rates, as described before (Figure 3C,D). Importantly, Western blot analysis indicated no substantial amounts of incompletely translated or degraded protein as well as comparable expression levels in case of the GlpF tetramers WT_3_/EA_1_, WT_2_/EA_2_ and WT_1_/EA_3_ (Figure 3E and Figure S3). Only the expression level of the fused GlpF tetramer EA_4_ was reduced by a factor of 1.8 ± 0.46 compared to the WT_4_ construct. As can be seen in Figure 3C,D, the activity of the fused tetramers decreases with the fraction of E43A-mutated monomers.

To gain information about a possible functional crosstalk between the GlpF monomers with the impaired E43A-variants in a fused tetramer, the measured rate constants of the ribitol conductance facilitated by the GlpF heterotetramers after induction with 500 µM IPTG were compared with the values expected for different scenarios. Due to a lower expression level (Figure 3E and Figure S3), the rate constant of the GlpF tetramer EA_4_ was corrected using a factor of 1.8 (k_uncorrected_ = 0.16 s^−1^ ± 0.082 and k_corrected_ = 0.29 s^−1^ ± 0.15). This correction factor as well as its calculated error stem from the different ratio of the EA_4_ expression level in comparison to the expression level of WT_4_ (see also Figure S3). It is noteworthy that as the accuracy of determining an expression level via Western blot analysis is limited, an overcorrection of the rate constants cannot be ruled out, and thus the channel activity might even be lower than calculated.

**Figure 4:**
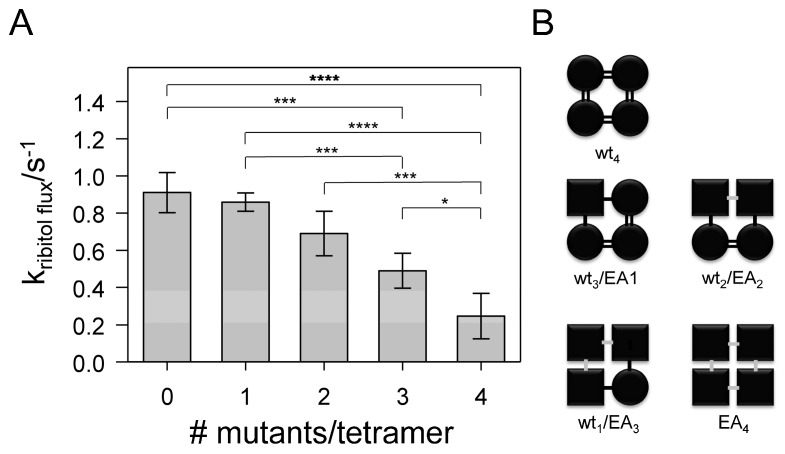
Impact of the number of EA-mutated protomers per tetramer on GlpF activity. (**A**) The measured rate constants of the fused GlpF tetramers plotted against the proportion of E43A-mutated monomers. The rate constants of fused GlpF tetramers are depicted as mean values (*n* = 3 ± SD). In the case of the fused heterotetramer EA_4_, the rate constant after normalization to the expression level is shown. Statistical analyses (one-way ANOVA, Tukey-test) yielded a *p*-value < 0.001 for the overall comparison. Statistically significant differences observed in a pairwise analysis are indicated in the figure: *p* < 0.001 (****), 0.001 < *p* < 0.01 (***), 0.05 < *p* < 0.1 (*). (**B**) Schematic depiction of interactions between individual GlpF monomers, with WT protomers having the strongest interactions (**=**), followed by weaker interactions between a WT and a mutated protomer (**-**) and weakest interactions between two mutated monomers (**-**).

The activity of covalently linked tetramers, containing increasing amounts of the mutated protomers, decreased with an increasing number of mutants per tetramer (Figure 4A).

In order to test the statistical significance of the observed activity differences, a one-way ANOVA test was employed. Based on this analysis, the probability that the differences observed within the five values occur by chance is less than 0.001 (*p* < 0.001). A number of pairwise comparisons resulted in *p*-values < 0.1 (Figure 4A).

The observed strong difference between the control (WT_4_) and EA_4_ could be explained either by an intrinsically lowered activity of the mutant or, alternatively, by a lack of proper protomer–protomer contacts. Based on the GlpF crystal structure [9], the mutated residue Glu43 points away from the monomer structure, thus this residue does not appear to be involved in stabilization of a GlpF monomer (see Figure 1). However, the results presented here are in agreement with unsuitable protomer–protomer contacts, since introduction of only one mutated monomer (protein WT_3_/EA_1_) did not lead to a significant reduction of activity when compared to WT_4_, indicating that contact of a mutated protomer with two WT protomers rescues WT activity. However, this stabilizing effect seemed to require WT protomers on both contact sides of a mutated protomer (Figure 4B). In the case of WT_2_/EA_2_ and WT_1_/EA_3_ proteins, the overall activity of the tetramers was reduced, possibly because mutant-WT contacts can be established only on one side of the mutated protomers (Figure 4B). Thus, here protomer–protomer interactions were not sufficiently robust to establish WT activity. However, when compared to EA_4_, the heterooligomer WT_2_/EA_2_ had a significantly increased activity.

**Figure 5:**
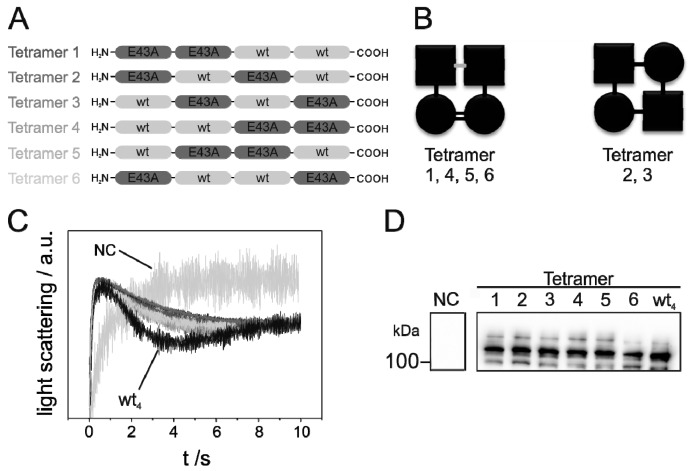
Activity of genetically fused GlpF heterotetramers containing two mutated protomers at different positions. (**A**) Schematic representation of the fused WT_2_/EA_2_ permutations. The light gray and dark gray boxes represent WT and E43A-mutated GlpF, respectively. (**B**) Schematic depiction of interactions between individual GlpF monomers, with WT protomers having the strongest interactions (**=**), followed by weaker interactions between a WT and a mutated protomer (**-**) and weakest interactions between two mutated monomers (**-**). (**C**) Typical light-scattering curves of the fused GlpF tetramers (different gray tones: compare (**A**)) and WT_4_ (black) after induction of protein expression with 500 µM IPTG. GlpF-free control cells (NC) (gray) were transformed with the plasmid pMalp2 and treated exactly as protein-expressing cells. The light-scattering curves are an average of five measurements. (**D**) Western blot analysis, determining the expression level of the studied permutated GlpF heterooligomers as well as the fused GlpF WT homooligomer (WT_4_). For the Western blot analysis, an antibody recognizing the GlpF C-terminus was used. See also Figure S4.

To further assess the specificity and promiscuity in monomer interactions mediated by Glu43, the activity of all possible permutations of the WT_2_/EA_2_ protein were determined. Five additional WT_2_/EA_2_ heterooligomer combinations were investigated that contained two E43A monomers at different positions (Figure 5A).

The activity of the mutated GlpF variants was again assessed by measuring the ribitol conductance rates (Figure 5C). The expression level was similar for WT_4_ and all WT_2_/EA_2_ variants (Figure 5D and Figure S4), and the determined rate constants were all about 0.7 s^−1^ with a SEM of 0.1–0.2 s^−1^. Thus, compared to WT_4_, the activity of all WT_2_/EA_2_ heterooligomers was reduced by ~28% compared to the fused GlpF WT-tetramer, in perfect agreement with the already observed results (Figure 4A). However, monomer–monomer contacts differed to some extent when tetramers 1, 4, 5 and 6 are compared to the tetramers 2 and 3 (Figure 5B). In the latter ones, every WT monomer was flanked by mutated monomers, whereas in the former two WT monomers interacted with each other and each with one mutated monomer. Furthermore, two mutated monomers interacted with each other. Thus, as the activities of all (permutated) fused tetramers were just about the same, weakened protomer interactions in tetramers 1, 4, 5 and 6, which all included a contact between mutated monomers, can likely be compensated (to some extent) by a “strong” WT–WT contact.

In summary, the results indicated that disturbed interactions between the monomers caused by mutation of Glu43 to Ala cannot be fully compensated by covalently linking the protomers within a tetrameric GlpF assembly, but contacts to and between WT protomers compensated weakened interactions to some extent.

## 3. Materials and Methods

### 3.1. Cloning and Mutagenesis

Fusion of the *glpF* gene was achieved via introducing restriction–digestion sites at the 5´and/or 3´end of the GlpF coding regions. For construction of fused *glpF* genes, the *glpF* gene was ligated one by one into the plasmids pRSet-His and pMalp2 (Table 1).

The *glpF* gene was amplified by PCR from the plasmid pGlpF (Table 1) [16] to introduce the restriction sites of the compatible enzymes *Xho*I and *Sal*I at the 5′- and 3′-end, respectively, using the primers GlpF_NdeI_XhoI_for and GlpF_SalI_rev (Table 2). Via this PCR, the stop codon at the 3′-end of the *glpF* gene was also removed. The purified PCR fragments were restriction-digested with NdeI/BamHI (New England Biolabs, Ipswich, MA, USA), and ligated into the equally restriction-digested plasmid pMalp2 (New England Biolabs, Ipswich, MA, USA). After ligation, a stop codon was inserted at the 3′-end of the *glpF* gene via site-directed mutagenesis, using the primers QC_GlpF_Stop_SalI_for and QC GlpF_Stop_SalI_rev to generate the plasmid p1xGlpF. The plasmid for expression of the homotetrameric GlpF fusion (WT_4_) was constructed by successive restriction–digestion and ligation. The *glpF* gene (without the stop codon) was restriction-digested with NdeI/SalI and ligated into the respective NdeI/XhoI restriction-digested vectors. For the expression of GlpF tetramers, containing different amounts of E43A-mutated GlpF (WT_3_/EA_1_, WT_2_/EA_2_, WT_1_/EA_2_ and EA_4_), the E43A mutation [16] was introduced into the *glpF* gene via site-directed mutagenesis. The WT *glpF* genes were successively fused first, and thereafter the mutated *glpF* genes. The DNA sequences were confirmed by DNA sequencing. The introduction of the compatible restriction–digestion sites caused the insertion of two amino acids (Val and Glu) in between monomers. As the GlpF C-terminus consists of 22 amino acids that are not part of TM helices, a longer linker sequence was not necessary. To facilitate the simultaneous NdeI/XhoI restriction–digestion of the plasmid, nine nucleotides were inserted between these restriction–digestion sites. Including the restriction–digestion sites, this resulted in an elongation of six amino acids at the N-terminus of the fusion protein (MGSGLE). The respective nucleotide sequences are given in Table 2.

### 3.2. GlpF Activity Measurements

The activity of the GlpF homo- and heterooligomers was assessed via measuring the flux of the polyalcohol ribitol across the *E. coli* inner membrane. After transformation with the pMalp2-based plasmids, the homo- and heterooligomeric proteins were expressed in *E. coli* SK46 cells (deficient in the two *E. coli* AQPs GlpF and AqpZ) in LB (lysogeny broth) medium containing 100 µg/mL ampicillin (Roth, Karlsruhe) and increasing IPTG (Roth, Karlsruhe) concentrations (0–500 µM) [28]. At an OD_600_ of ~0.6, the cell density was adjusted to OD_600_ = 1.0 in LB medium containing 100 µg/mL ampicillin and 30 µg/mL chloramphenicol (Roth, Karlsruhe) to avoid further cell growth. The protein activity was assessed using a SX20 Stopped-Flow Spectrometer (Applied Photophysics, Leatherhead, UK) by rapidly mixing the cell suspension with an equal volume of LB medium containing 600 mM ribitol (Alfa Aesar, Heysham, UK), 100 µg/mL ampicillin and 30 µg/mL chloramphenicol (Roth, Karlsruhe). Ribitol was chosen as the substrate to reduce the background flux in absence of GlpF. While the ribitol conductance rate of GlpF was comparable to the glycerol conductance rate, the intrinsic permeability of membranes for ribitol was significantly lower than for glycerol [9]. The light-scattering intensity was measured at 25 °C at a wavelength of 600 nm in a 90° angle. Due to water efflux, the scattering intensity quickly rose upon mixing, immediately followed by a decay caused by the ribitol influx. This decay was analyzed using a single exponential decay function to determine the rate constant *k* of GlpF-mediated ribitol flux.

### 3.3. Isolation of GlpF from Membranes and Western Blot Analysis

To determine the amount of expressed protein, GlpF-containing membranes were isolated from the *E. coli* SK46 cells used for the activity measurements. Cells were grown in LB medium containing 100 µg/mL ampicillin and IPTG concentrations between 0 and 500 µM. After reaching an OD_600_ of 0.8, cells were centrifuged (10 min, 3.220 g, 4 °C) and resuspended in 25 mM Tris-HCl (pH 8.0), 2 mM Na_2_EDTA × 2H_2_O and 0.1% (*v*/*v*) protease inhibitor cocktail, thereby adjusting the OD_600_ to 2.0 in a volume of 15 mL. Cells were then disrupted by sonication in an ice-water bath, using the Branson Sonifier 250 (G. Heinemann, Schwäbisch Gmünd). Cell debris was removed by centrifugation (12,000× *g*, 4 °C, 10 min), and GlpF-containing membranes were collected by ultracentrifugation (165,000× *g*, 4 °C, 1 h). The membrane pellet was resuspended in 100 µL 50 mM phosphate buffer (pH 8.0), 300 mM NaCl and 10% glycerol. The protein concentration of the membrane fraction was determined via a bicinchoninic acid assay, using the BCA protein assay kit (Thermo Fisher Scientific, Rockford, IL, USA).

Membranes with a total protein concentration of 1.2 µg were incubated in an SDS-PAGE sample buffer (2% (*w*/*v*) SDS, 50 mM dithiothreitol (DTT), 50 mM Tris-HCl (pH 6.8), 10% (*v*/*v*) glycerol and 0.04% (*w*/*v*) bromphenol blue) for 15 min at room temperature. After performing the SDS-PAGE analysis on 10% SDS-PAGE gels, the separated proteins were transferred to a polyvinylidene difluoride (PVDF) membrane, and thereafter GlpF was detected using an antibody directed against the GlpF C-terminus (VVEEKETTTPSEQKASL, Gramsch Laboratories, Schwabhausen) [16]. The relative expression levels of the GlpF constructs (relative to the WT protein) were determined by densitometry using the program ImageJ [29].

### 3.4. Purification and SDS-PAGE Analysis

To determine the stability of WT GlpF as well as GlpF E43A, GlpF membranes were solubilized in dodecyl-β-D-maltoside (DDM) and GlpF was purified as described in detail in [30]. For SDS-PAGE analysis, no additional SDS was present in the SDS-PAGE sample buffer (50 mM Tris-HCl (pH 6.8), 10% (*v*/*v*) glycerol and 0.04% (*w*/*v*) bromphenol blue) to preserve the tetrameric state of GlpF. As a protein standard, the Pierce™ Unstained Protein MW Marker (Thermo Fisher Scientific, Rockford, IL, USA) was used.

The PDB-ID of the GlpF structure shown in the graphical abstract is 1FX8.

## 4. Conclusions

Monomer–monomer contacts drive GlpF tetramerization and enhance its channel activity, most likely via inducing subtle structural adjustments and stabilization of an active GlpF monomer structure. Our previous analyses indicated that Glu43 is of special importance for assembly and/or stability of GlpF tetramers. The here presented analyses of the various genetically fused GlpF oligomers now suggest that the defects observed after mutation of Glu43 might be compensated to some degree via covalently linking the monomers, albeit the covalent linkage cannot fully compensate for defective protomer–protomer interactions. This supports the assumption that defined protomer–protomer interactions, as well as spatial proximity, are crucial for the channel activity of individual GlpF monomers within the tetrameric assembly. Potential inter-subunit stabilization likely involves several different contact sites within the monomer besides interaction of Glu43, as permuting the WT_2_/EA_2_ protein did not result in altered channel activities.

As the tertiary and quaternary structures of AQPs are highly conserved, our results likely apply to other AQPs as well: Solely the quaternary structure of AQPs, involving defined interactions of individual protomers, ensures proper activity of AQP channels.

## Figures and Tables

**Table 1:** Plasmids used in this study. All plasmids carry an ampicillin resistance cassette.

Plasmid	Reference
pGlpF	[16]
pMalp2	[16]
p4xGlpF	This study
p1xGlpF-E43A	This study
p3xGlpF-1xGlpF-E43A	This study
p2xGlpF-2xGlpF-E43A	This study
p1xGlpF-3xGlpF-E43A	This study
p4xGlpF-E43A	This study
p2xGlpF-2xGlpF-E43A tetramer 2	This study
p2xGlpF-2xGlpF-E43A tetramer 3	This study
p2xGlpF-2xGlpF-E43A tetramer 4	This study
p2xGlpF-2xGlpF-E43A tetramer 5	This study
p2xGlpF-2xGlpF-E43A tetramer 6	This study
pRSET-His-GlpF	[16]

**Table 2:** Oligonucleotides used in this study. *glpF* sequences are underlined and sequences recognized by restriction enzymes are highlighted in gray. Mutated and added bases are highlighted in bold.

Primer	5′-Sequence-3′
GlpF *Nde*I *Xho*I for	GCGCGCCATATGGGCAGCGGCCTCGAGATGAGTCAAACATCAACC
GlpF *Sal*I rev	GCGCGCGGATCCGTCGACCAGCGAAGCTTTTTG
QC GlpF Stop SalI for	CAAAAAGCTTCGCTG *TAA* GTCGACGGATCCGGC
QC GlpF Stop SalI rev	GCCGGATCCGTCGAC *TTA* CAGCGAAGCTTTTTG
QC GlpF-E43A for	CGTCTTTTGGTCAGTGGG *C* AATCAGTGTCATTTGGG
QC GlpF-E43A rev	CCCCAAATGACACTGATT *G* CCCACTGACAAAAGAC

## Supplementary Materials

Supplementary materials can be found at http://www.mdpi.com/1422-0067/20/4/927/s1.

## Abbreviations

AQP	aquaporin
WT	wild-type
T	tetramer
NC	negative control
IPTG	isopropyl β-D-1-thiogalactopyranoside
M	molecular mass
TM	transmembrane
*E. coli*	Escherichia coli
GlpF	glycerol facilitator
D	Dimer
Tr	Trimer
