# Supplementary material for: Covalently Linking Oligomerization-Impaired GlpF Protomers Does Not Completely Re-establish Wild-Type Channel Activity
Source: Int J Mol Sci. 2019 Feb 20;20(4):927. doi: 10.3390/ijms20040927 (PMC6412381; doi:10.3390/ijms20040927)
Supplement: Supplementary file 1 [file ijms-20-00927-s001.pdf]

Supporting information

Covalently Linking Oligomerization-Impaired GlpF Protomers Does Not Completely Re-establish Wild-Type Channel Activity

Noreen Klein[1,†], Margareta Trefz[1,†] and Dirk Schneider [1,*]

[1] Johannes Gutenberg-University Mainz, Institute of Pharmacy and Biochemistry, Johann-Joachim-Becher-Weg 30, 55128 Mainz, Germany; noreen-klein@freenet.de (N.K.); martrefz@uni-mainz.de (M.T.)

[*] Correspondence: Dirk.Schneider@uni-mainz.de; Tel.: +49 6131 39-25833 (D.S.)

† These authors contributed equally

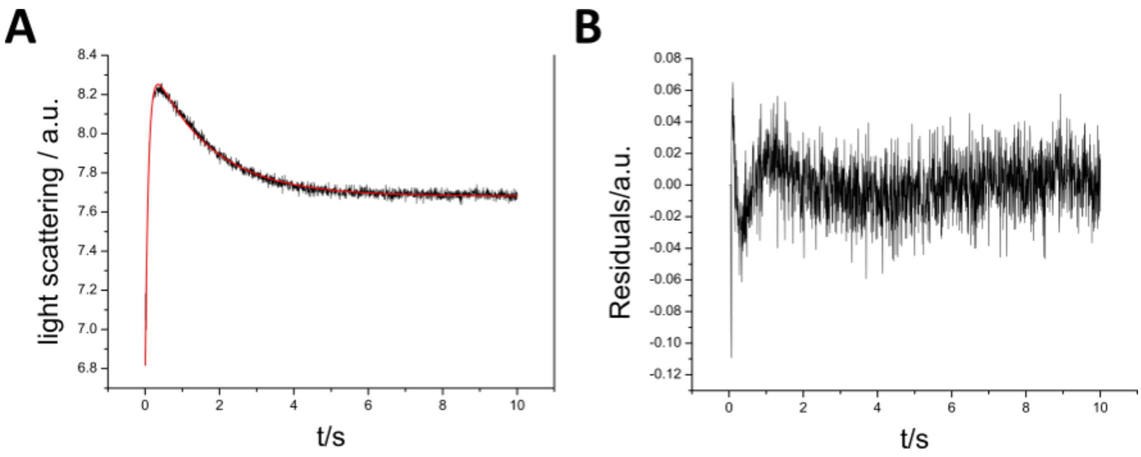

Figure S1: Light-scattering curve fitting.
(A) An example of a light-scattering curve is shown in black. Rapid mixing of GlpF with ribitol results in fast cell shrinking, visible as a steep increase of the light-scattering signal. The ribitol and water influx through GlpF is visible as the slow decay in light-scattering intensity. The applied single exponential fit is shown in red. (B) Distribution of residuals of the fit function shown in (A).

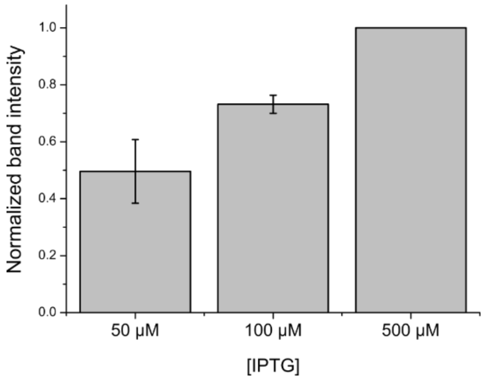

Figure S2: Densitometric analyses of the Western blots used for the determination of the wt_4 expression level at increasing [IPTG] (compare Figure 2 in the main text). The expression level of membrane-integrated GlpF increases with increasing [IPTG]. The intensity at 500 µM IPTG was set as 1.0 ($n = 3 \pm SD$).

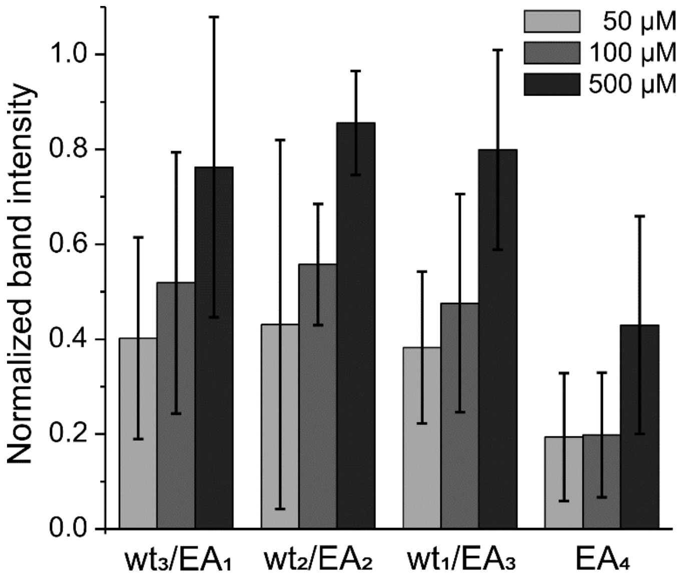

Figure S3: Densitometric analyses of the Western blots used for quantification of the expression level of fused GlpF heterooligomers (compare Figure 3 in the main article). The expression level of membrane-incorporated GlpF increases with increasing [IPTG] (different IPTG concentrations are shown in different gray tones). The intensity of the wt_4 construct at [IPTG] = 500 µM was set as 1.0. Note that the SD values are high at 50 and 100 µM IPTG. At [IPTG] = 500 µM, most heterooligomers show similar expression levels. Solely the expression level of EA_4 is reduced by 1.8 in comparison to wt_4 ($n = 3 \pm SD$).

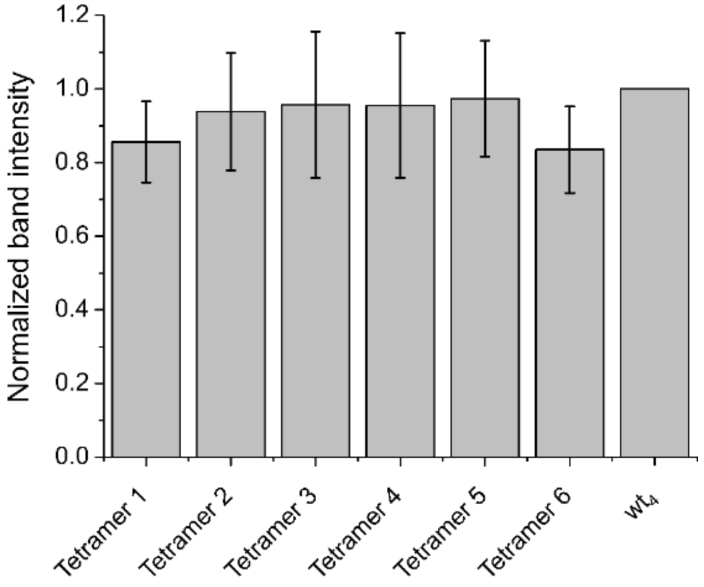

Figure S4: Densitometric analyses of the Western blots used for the determination of the expression level of the fused GlpF wt_2/EA_2 permutations (compare Figure 5 in the main article). All produced heterooligomers have an expression level that is comparable to the GlpF wt_4 homooligomer, which was set as 1.0 ($n = 3 \pm SD$).
